# Benchmarks for Evidence-Based Risk Assessment with the Swedish Version of the 4-Item Psychosocial Safety Climate Scale

**DOI:** 10.3390/ijerph17228675

**Published:** 2020-11-22

**Authors:** Hanne Berthelsen, Tuija Muhonen, Gunnar Bergström, Hugo Westerlund, Maureen F. Dollard

**Affiliations:** 1 Centre for Work Life and Evaluation Studies (CTA), Malmö University, 205 06 Malmö, Sweden; tuija.muhonen@mau.se; 2 Section 4, Faculty of Odontology, Malmö University, 205 06 Malmö, Sweden; 3 Department of School Development and Leadership, Faculty of Education and Society, Malmö University, 205 06 Malmö, Sweden; 4 Centre for Musculoskeletal Research, Department of Occupational Health Sciences and Psychology, University of Gävle, 801 76 Gävle, Sweden; gunnar.bergstrom@hig.se; 5 Unit of Intervention and Implementation Research for Worker Health, Institute of Environmental Medicine, Karolinska Institute, 171 77 Stockholm, Sweden; 6 Department of Psychology, Stress Research Institute, Stockholm University, 106 91 Stockholm, Sweden; hugo.westerlund@su.se; 7 PSC Observatory, Centre for Workplace Excellence, Justice and Society, University of South Australia, Adelaide, SA 5001, Australia; maureen.dollard@unisa.edu.au

**Keywords:** psychosocial safety climate, PSC-4, occupational safety and health, OSH, risk assessment, benchmark, COPSOQ, Sweden

## Abstract

The purpose of the present study was to validate the short version of The Psychosocial Safety Climate questionnaire (PSC-4, Dollard, 2019) and to establish benchmarks indicating risk levels for use in Sweden. Cross-sectional data from (1) a random sample of employees in Sweden aged 25–65 years (*n* = 2847) and (2) a convenience sample of non-managerial employees from 94 workplaces (*n* = 3066) were analyzed. Benchmarks for three PSC risk levels were developed using organizational compliance with Occupational Safety and Health (OSH) regulations as criterion. The results support the validity and usefulness of the Swedish PSC-4 as an instrument to indicate good, fair, and poor OSH practices. The recommended benchmark for indicating good OSH practices is an average score of >12.0, while the proposed cutoff for poor OSH practices is a score of ≤8.0 on the PSC-4. Scores between these benchmarks indicate fair OSH practices. Furthermore, aggregated data on PSC-4 supported its reliability as a workplace level construct and its association with quantitative demands, quality of leadership, commitment to the workplace, work engagement, job satisfaction, as well as stress and burnout. Thus, the Swedish version of PSC-4 can be regarded as a valid and reliable measure for both research and practical use for risk assessment at workplaces.

## 1. Introduction

Numerous studies and reports during the last decades have pointed out work-related stress as a serious global problem [1], leading to harmful consequences and costs for individuals, organizations, and societies [2,3,4,5]. Today, it is the risks in the psychosocial work environment that are increasing and prove difficult to handle [6]. Compared to traditional safety and health risk management, largely focusing on physical risk factors, there is a lack of procedures for dealing with psychosocial risk management [2]. Furthermore, work-related stress is often regarded as a problem of the individual employees, who are recommended to engage in personal stress-relieving activities such as mindfulness, meditation, and resilience training [7,8]. This may imply that an individual who gets stressed is regarded as weak and failing whereas organizational measures in order to change the working conditions are not put forward.

In contrast to this approach, Dollard and Bakker developed the theory of Psychosocial Safety Climate (PSC) [9]. They consider PSC as a property of the organization and define it as ‘‘policies, practices, and procedures for the protection of worker psychological health and safety’’ (p. 580). The PSC manifests the true priorities of an organization and emphasizes the senior management’s commitment and priority given to employees’ psychological well-being and security [9]. Thus, in organizations with high PSC, the managers give a higher priority to work conditions that protect and enhance employee psychological health instead of solely focusing on productivity [10]. According to the theory, PSC is regarded as an upstream organizational factor predicting work-related psychosocial risks, e.g., demands and resources [9], and is therefore referred to as the “cause of the causes” of work stress [11,12].

In Sweden, the Occupational Safety and Health (OSH) regulations were revised in 2015 based on reports of a steady increase of stress-related long-term sick leave. The new provisions focus on organizational and social aspects in the work environment [13]. This implies that, instead of focusing on individual causes (“blaming the victim”), the provisions specifically clarify the obligations of employers to systematically evaluate and address problematic issues in the work environment in an active dialogue with both the employees and the health and safety representatives. Thus, the Swedish provisions are in line with the theory of PSC, which similarly focuses on the responsibility of organizations and senior managements (rather than of the individual employees) to prevent stress at work by designing psychologically safe workplaces [10].

### 1.1. Assessing the Psychosocial Safety Climate

In order to assess the employees’ perception of the organizational policies, practices, and procedures concerning employee psychological health, the Psychosocial Safety Climate scale (PSC-12) was developed by Hall et al. in 2010 [14]. PSC-12 consists of four theoretically based dimensions: how employees perceive that senior management (1) engages, (2) prioritizes, (3) communicates with, and (4) involves employees in handling psychosocial workplace safety issues [9,14].

In several studies, PSC has been integrated with the Job Demands-Resources (JD-R) Model [15,16] both as a predictor of health erosion and work motivation paths and as a moderator between job demands and their effect on health and safety outcomes [9,17]. A vast number of empirical studies conducted during the past ten years provide convincing evidence for PSC as an important indicator of OSH practices and working conditions, low PSC being related to high job demands, low job resources, higher effort–reward imbalance, as well as bullying and harassment [17,18,19].

So far, the majority of the PSC studies have been conducted in Australia and Malaysia [17,20], but recently, the PSC scale has been translated and validated in the European context [21,22,23,24]. While validating PSC-12 in the Swedish and German contexts, issues of overlapping items were raised, calling for a shorter (less redundant) version of the scale [21,22]. As workplace surveys often need to cover several topics besides PSC, short reliable and valid measures are needed in order to reduce participants’ response burden and to ensure adequate response rates. Thus, a parsimonious version of the scale, PSC-4, has been introduced, consisting of one item from each of the theoretically based four domains described above [9,25]. The results using three time-lagged Australian samples demonstrated satisfactory predictive validity and reliability of the PSC-4 [25], but more studies in different organizational and cultural contexts are needed for further empirical testing of the ultrashort measure [10].

### 1.2. The Context of the Current Study

Women and men in Sweden participate in the labor force approximately to the same extent, but the Swedish labor market remains highly gender segregated regarding the sectors, occupations, and hierarchical positions that women and men work in [26]. Women work to a larger extent in the public sector and in human service organizations (e.g., schools, and social and health care) and are underrepresented as managers. Earlier research has shown differences in organizational conditions depending on the gendered context, managers in female-dominated organizations having fewer resources, less support, and larger span of control, i.e., a higher number of subordinates [27,28], and a lack of access to hierarchical networks than managers in male-dominated organizations [29]. To our knowledge, neither gender nor span of control have been investigated in earlier PSC studies. We want to address this knowledge gap as it seems relevant considering the gender-segregated labor market in Sweden and further as there are indications that PSC can vary independently on different hierarchical positions suggesting multiple PSC realities [30].

Bailey and Dollard have pointed to a need for research to determine the levels of PSC in countries other than Australia to set standards for work quality and worker health [31]. While the Australian benchmarks were determined based on the risk of depression and work strain [31,32], the current study will establish benchmarks using a criterion-based approach. Loh et al. discussed the distinction between espoused and enacted PSC [33]. The policies, practices, and procedures reflect the espoused PSC, i.e., the pronounced intentions of the senior management, whereas beyond rhetoric, the enacted PSC refers to the concrete actions taken by the lower level managers, i.e., what measures they have taken in line with the policies. It can therefore be argued that PSC measures should be combined with other measures in order to capture the enacted PSC. In this study, we apply perceived Occupational Safety and Health (OSH) practices (as an indicator for enacted PSC) when establishing risk levels of PSC-4.

Even though PSC is considered as a function of the organization and evident as shared perceptions, earlier studies have assessed PSC at the individual, work group, and organizational levels [17,20]. According to Dollard and Bailey, PSC has an effect both at the work group and individual levels [12]. Dormann et al. also emphasize the need for more knowledge of PSC both as an upstream and downstream factor, as different PSCs might exist at the different organizational levels [19]. In the current study, we establish benchmarks at the individual level (national random sample) and thereafter apply the benchmarks at the workgroup level (workplace sample).

As several studies have integrated PSC with the JD-R model (c.f. [17]), we also assessed demands and resources by using scales from Copenhagen Psychosocial Questionnaire (COPSOQ) [34,35] in order to analyze the applicability of the established PSC-4 benchmarks.

The overall purpose of the present study is to validate the 4-item version of PSC and to establish national benchmarks indicating risk levels of PSC for use at Swedish workplaces. The first aim is to apply a criterion-based approach based on staff-reported workplace practices related to the Swedish legislation on organizational and social work environment for identifying risk levels of PSC-4. The second aim is to evaluate how the PSC-4 risk levels are associated with quantitative demands, leadership quality, job satisfaction, commitment to the workplace, work engagement, burnout, and stress both at the individual level and at aggregated workplace levels.

## 2. Materials and Methods

The present study builds on cross-sectional data (1) from a national random sample survey for establishment of benchmarks and (2) from a convenience sample of employees from 94 workplaces for cross validation at the organizational aggregated level.

### 2.1. Random Sample of Swedish Working Population

A random sample of 11,556 inhabitants in Sweden aged 20–65 years and registered as gainfully employed was drawn from the Swedish employment directory. Data was collected by Statistics Sweden (SCB) by post, including an information letter, a paper questionnaire together with a stamped return envelope, and a personal link to a web version of the questionnaire. Non-respondents received up to two reminders, the last of these included new paper questionnaires and return envelopes. Data collection took place from September to November 2018. In total, 3642 responded (30.9%). In general, women, the oldest age group, and those with tertiary education were the most likely to respond. People born in Scandinavia were more likely to respond than those born elsewhere, and those with the highest income responded to a larger extent than others. Inclusion criteria for the present study were 25–65-year-old workers living in Sweden, gainfully employed during the last 3 months before the survey, and having a superior/colleagues. This led to a final database for analyses *n* = 2847 as previously reported in more detail [34].

### 2.2. Workplace Sample

Cross-sectional questionnaire data was collected in 2017–2020. All staff members at a convenience sample of 94 workplaces (32 private, 40 public, and 22 from the non-profit sector; 42 workplaces were in human service organizations) received an email with a link to an online questionnaire and information about the research project. We defined workplace as geographically separate units where people conduct their daily work and share the same local management. Workplaces with less than 5 respondents were excluded from the study. Each survey was open 3–4 weeks and included two reminders. The average response rate for the sample of workplaces was 79%, and analyses included data from 3066 non-managerial employees. The average number of respondents at the workplaces was 33 (SD 29, Range 5-153). For this convenience sample, 28% of the employees were under 35 years of age, 25% were 35–44, 25% were 45–54, and 22% were aged 55 or older, and 64% were women; 89% of the respondents had relational work (direct contact to clients, patients, customers, etc. in their work). The corresponding distribution for the target population 2017 was according to Statistics Sweden: 26% below age 35, 26% were 35–44 years old, 28% were 45–54, and 21% were 55 or older, and 48% were women.

### 2.3. Variables

The questionnaire for the national sample comprised 132 items in total and a free text field for comments. In the present study, we included the following demographic background factors: gender, age, work sector, weekly working hours, relational/non-relational work, position, kind of employment, normal work time, and size of local workplace measured by span of the nearest leader.

PSC was measured by 4 items with 5 response options [14,36] in the Swedish version [21,37].

OSH practices were measured by 5 items relating to the Swedish legislation and regulations [13]. Of these, 4 items were from the Labor Inspection’s work environment survey from 2017 [38] and 1 was an additional item. The response option “Yes” was considered to reflect the ideal situation, “No” was a problematic situation, and “Don’t know” was in the middle based on findings from a cognitive pretesting of items prior to the survey [39]. The exact formulations are presented in Appendix A, Table A1.

The following scales from the Swedish standard version of COPSOQ III [34,35] were included: quantitative demands (3 items, example: Do you get behind with your work?), quality of leadership (3 items, e.g., To what extent would you say that your immediate superior is good at solving conflicts?), commitment to the workplace (3 items, e.g., How often do you consider looking for work elsewhere?), job satisfaction (4 items, e.g., Regarding your work in general, how pleased are you with your job as a whole, everything taken into consideration?), stress (3 items, e.g., How often have you had problems relaxing during the last 4 weeks?), and burnout (3 items, e.g., How often have you felt worn out during the last 4 weeks?).

Work engagement was assessed by 3 items (e.g., At my work, I feel bursting with energy), the adapted COPSOQ-version of work engagement, originating from the Utrecht Work Engagement Scale [40]. All the scales above had 5 response options on a Likert scale.

From the workplace surveys, we included the same PSC and COPSOQ III items in addition to the background factors (gender, age, work sector, position, size, and kind of workplace).

### 2.4. Analyses

A scale (range 4–20) was created for PSC-4. The scale score was set to missing if respondents had replied to less than half of the items included in the scale. Weights were calculated for the nationally representative sample to match the target population based on gender, age, income, and educational level. The weighted mean for PSC-4 was calculated for the total sample for establishing a national average level. Unweighted mean scores and standard deviations were calculated for subgroups of respondents based on individual and work-related characteristics in order to analyze the impact of background factors. Differences in mean scores for PSC-4 between groups were analyzed by t-test including Levene’s Test for Equality of Variances and by 1-way ANOVA with Tukey HSD post hoc test for multiple comparisons. Separate linear multiple regression models were built for men and women with background factors (relational work, position, and sector dichotomized and three groups for increasing span of nearest leader) regressed on PSC.

A principal factor analysis with oblique rotation (direct oblimin) was run with the four PSC items and the five items OSH-practice items (unweighted data, non-managerial employees from the national sample). Inspection of the correlation matrix revealed that all items had at least one correlation coefficient greater than 0.30, with none greater than 0.90 (Appendix A, Table A2). The overall Kaiser–Meyer–Olkin (KMO) was 0.87, and Bartlett’s test of sphericity was statistically significant (*p* < 0.001), indicating the data was factorizable. The analysis revealed two components with eigenvalues greater than one. The two factors explained 46.4% (reflecting OSH practices), and 18.7% (reflecting PSC), respectively. The factor loadings from the two factors after rotation are shown in Appendix A, Table A3. The correlation between the two extracted factors was −0.38. The analysis corroborated that the OSH items and the PSC items reflect two related but distinct concepts.

A criteria-based approach was used for classifying different OSH practices into good–fair–poor levels [39], based on consensus in the research group. For an overview of response profiles, see Appendix B, Table A4. ANOVA tests were used for analyzing differences in mean values for PSC-4 depending on OSH practices. The benchmarks for PSC risk levels were determined based on PSC mean scores for good, fair, and poor OSH practices (non-managerial employees).

The scales quantitative demands, quality of leadership, job satisfaction, commitment to the workplace, stress, and burnout based on the Swedish standard version of COPSOQ III were computed as means of items with range 0–100, where the scale score was set to missing if respondents had replied to less than half of the items included in the scale (34, 35). Each scale was scored in the direction indicated by its name [34,35]. ANOVA tests including Levene’s Test for Equality of Variances and with Tamhane’s T2 post hoc test for multiple comparisons were used for analyzing differences in mean values of the scales depending on PSC risk level. For COPSOQ scales, a 5–10 point mean score difference is considered a minimum important difference [41].

Based on data from the workplace sample, the PSC-4 and COPSOQ III scale scores were aggregated from individual level data to workplaces to reflect the practical use of these instruments for evidence-based risk assessment. ICC(1) and ICC(2) were calculated. ICC(1) represents the amount of variance in the employees’ responses that can be explained by their membership of a group (workplace) [42,43,44,45]. ICC(1) values of 0.05 can be considered as a small to medium effect, and higher values indicate stronger effects, i.e., a larger proportion of the variance explained by the workplace [45]. ICC(1) values from applied field research of organizations is typically up to a maximum of 0.20 (p 362 in [43]). ICC(2) is an estimate of reliability of the aggregated group means [42,43,44]. Values <0.5 indicate poor reliability, 0.5–0.75 is moderate, and >0.75 indicates good reliability of group-level means [46]. Corresponding to the analyses at individual level, ANOVA tests were used for analyzing differences in mean values aggregated to workplace level of quantitative demands, quality of leadership, job satisfaction, work engagement, commitment to the workplace, stress, and burnout depending on PSC risk level.

### 2.5. Ethics

Informed consent was obtained from all individual participants included in the study. All procedures performed were in accordance with the ethical standards of the national research committee and with the 1964 Helsinki Declaration and its later amendments or comparable ethical standards. The Regional Ethics Board in Southern Sweden approved the study (Dnr 2015-476; 2018-392; 2019-05904).

## 3. Results

### 3.1. Random Sample of Swedish Working Population

Out of the 2847 respondents, 56% were women, and almost half of the respondents worked in the private sector (47%). Two out of three were in a non-managerial position (67%), and most respondents (81%) reported having direct contact with patients, customers, clients, pupils, etc. at work. The majority worked day hours between 06:00–18:00 h (78.5%). The size of the workgroup (measured by the control span of the nearest leader of the respondent) was up to 10 people for 37% of respondents, 11–20 people for 25%, and more than 20 people for 35% (Table 1).

The weighted mean score for PSC-4 was 11.5, with a standard deviation of 4.1 and the median 12.00. Scale missing was 1.1%, skewness was −0.06, kurtosis was −0.50, and Cronbach’s alpha was 0.93 (analyses based on weighted data). Item response distribution is presented in Appendix C, Table A6.

Bivariate analyses showed statistically significant differences in PSC-4 mean scores between subgroups of respondents based on individual as well as work characteristics (Table 1). Women reported a lower PSC-4 score than men did. Respondents working in private sector and those working day hours reported a higher PSC-4 mean score than others. The more managerial responsibility in the position and the smaller the size of the span of the nearest leader, the higher the reported PSC-4 score. No differences were found in relation to age, weekly work hours, and relational versus other kind of work or in relation to kind of employment (fixed, temporary, and hourly).

The regression analyses revealed that position and span of nearest leader were associated with PSC-4 for both men and women (Table 2). Managers reported higher PSC than other employees, and the larger the control span of nearest leader, the lower the PSC. A difference in relation to work sector was also found. Men working in the public sector experienced a higher PSC compared to men in the private sector. For women, the tendency was reversed, though not significant (*p* = 0.08).

The criteria-based classification approach resulted in 47% of the non-managerial respondents from the national sample being categorized as having a workplace with good OSH practices, 35% with fair practices, and 18% with poor OSH practices (Appendix B, Table A4 and Table A5). The mean score of PSC-4 differed significantly for the three levels of OSH practices (*p* < 0.001), and based on these findings, the benchmarks for PSC-4 risk levels were established (Table 3). A PSC score at 8 or lower indicates a need for urgent actions (red risk), higher than 8 and up to 12 indicates a need to pay more attention to regulations (yellow risk), and a score higher than 12 indicates a good level of enacted OSH practices (green risk). Out of 1903 non-managerial employees in the national sample, 27% was classified as having high PSC-risk level (red), 39% was classified as moderate PSC-risk level (yellow), 34% was classified as low PSC-risk level (green), while 1% was not possible to categorize due to internal missing values. 

Table 4 shows that the mean scores of the standard version of the COPSOQ III scales for quantitative demands, quality of leadership, job satisfaction, commitment to the workplace, work engagement, stress, and burnout differed significantly between the three PSC risk levels. The largest differences in raw mean scores (scale range 0–100) were found for quality of leadership (35 point) and commitment to the workplace (35 point), while the lowest differences were found for quantitative demands (12 point). For all scales, the difference between those categorized as working in a high in contrast to in a low PSC-risk environment exceeded the COPSOQ-criteria of 5–10 point, indicating a minimum important difference [34]. For the moderate risk level, all mean scores differed less than 5 point from the national benchmark for the COPSOQ scales and, correspondingly, all mean scores differed by more than 5 point in a positive direction for the low risk level and more than 5 points in a negative direction for the high risk level.

### 3.2. Workplace Sample

Table 5 displays the measures relating to aggregation of data to workplace level. The ICC(2) scores indicate a moderate to good reliability of group mean scores for workplaces. The ICC(1) scores, explaining the effect of respondents’ workplace, showed a strong effect for PSC-4, quantitative demands, quality of leadership, and commitment to the workplace; a small to medium effect was seen for job satisfaction, stress, and burnout; and no effect was found for work engagement (ICC(1)). The aggregated workplace mean scores of quantitative demands, quality of leadership, job satisfaction, commitment to the workplace, stress, and burnout differed significantly between the three PSC risk levels, corresponding to the results from the individual level data based on a random sample. The differences in mean scores between PSC risk levels were significant (*p ≤ *0.05) and exceeded the COPSOQ-criteria of 5–10 point, indicating a minimum important difference for all scales except for work engagement and between low and moderate PSC risk levels for quantitative demands.

**Figure 1:**
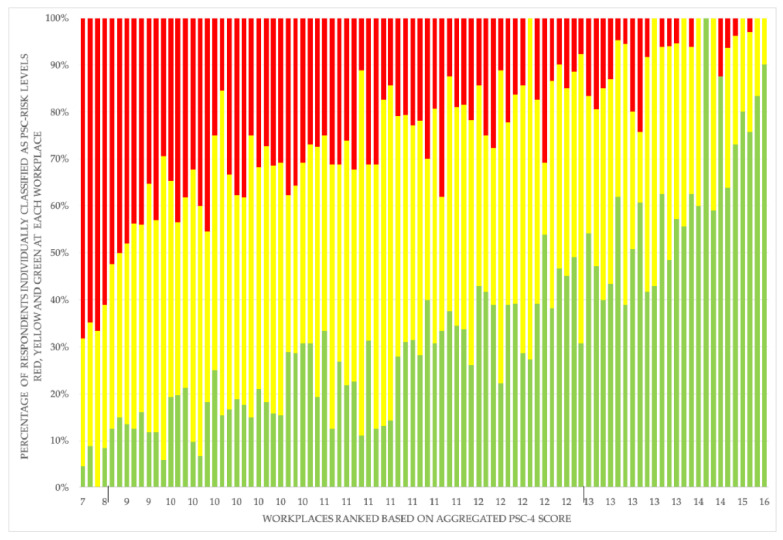
PSC-4 score aggregated to workplace level (*X*-axis) by percentage of respondents at the workplace who at the individual level have a low (green), moderate (yellow), and high (red) PSC-risk level (*Y*-axis): based on data from the workplace sample (*n* = 3066 non-managerial employees from 94 workplaces).

Figure 1 illustrates that most workplaces have individual employees indicating high, moderate, and low risk levels. However, the general tendency is that the proportion of individuals at high risk decreases with increasing PSC aggregated mean for workplaces.

## 4. Discussion

### 4.1. Main Findings

The results of the current study support the validity and usefulness of the Swedish version of PSC-4 as an instrument for identifying workplaces having poor, fair, and good OSH practices. PSC scores 8 or lower indicate poor OSH practices that might need urgent actions; scores higher than 8 and up to 12 are classified as fair, suggesting more attention should be paid for OSH practices; and scores higher than 12 indicates a good level of OSH practices. Further, concurrent validity of the three risk levels in relation to central aspects of work environment, strain, and motivational outcomes was corroborated. In sum, the current study confirms the Swedish version of PSC-4 as being a valid and reliable measure for both research and practical use for risk assessment at workplaces.

### 4.2. PSC in Relation to Background Factors

The average level of PSC was higher for managers, especially for those with staff responsibility, than for other employees. This is in line with findings from a study on PSC among police officers [30]. At the workplace level, differences in perceptions of psychosocial risks call for a social dialogue between managers and employee representatives for promoting psychosocial risk management [47]. Previous research has shown that PSC works invariantly in relation to related constructs for public and private sector employees, but it has not analyzed differences in PSC levels between sectors [48]. In the present study, private sector employees on average reported higher PSC than public sector employees. However, this result was confounded by gender as men from the public sector rated PSC higher than men from the private sector. A consistent finding among both women and men was that the more subordinates the nearest leader had, the lower the average level of PSC. In Sweden, the public sector employs around 1.5 million people and the majority (circa 70%) is women [26]. While women in the public sector mainly work within care, social work, and schools, the men are employed in more technical jobs [27]. The managerial structures differ so that managers in the female-dominated jobs have a larger span of control than managers in the male-dominated jobs [28]. As a line manager, being able to enact good PSC leadership requires, besides commitment and support from senior management, necessary prerequisites, including a reasonable span of control.

### 4.3. Benchmarks Indicating PSC Risk Levels

The national benchmarks indicating PSC risk levels at Swedish workplaces are lower than the Australian ones [31]. The Australian benchmarks are, for low-risk PSC, >13.7; for moderate risk, 12.3–13.7; and for high risk, <12.3 (risk levels reported on a scale 12–60 converted to 4–20) [31]. If we had applied the Australian benchmarks, 66% of the Swedish national sample would have been categorized as having a high risk and only 26% would have been considered at low risk. The overall national average of PSC-4 was also lower for the Swedish than for the Australian populations (11.5 for Sweden compared to 13.2–13.4 reported for the years 2009–2015 from the Australian Workplace Barometer study (p 393 in [36])). While the difference between Australian and Sweden regarding cutoff for low risk corresponds to the difference in population averages, the difference is larger concerning a high risk. In other words, the interval for moderate risk is larger in Sweden than in Australia. This lack of proportional correspondence between cutoff values for risk levels could be expected since the present Swedish study used different external criteria for developing the benchmarks than the Australian study [31]. However, the remarkably lower average level of PSC in Sweden compared to Australia calls for a need for further cross-cultural validation including analyses of measurement invariance across international translations of the instrument.

In the present study, we analyzed concurrent validity of PSC risk levels in relation to constructs measured by the COPSOQ questionnaire, i.e., quantitative demands, quality of leadership, job satisfaction, commitment to the workplace, work engagement, stress, and burnout. An earlier version of COPSOQ was also used in one of the first Australian studies placing PSC in its nomological framework [9]. Our approach was to evaluate how the PSC risk levels related to the Swedish national benchmarks for COPSOQ scales. We found solid support for the established PSC risk benchmarks for use in the Swedish context, both at the individual and workplace levels. First, the mean values of the investigated COPSOQ scales were close to the Swedish national COPSOQ benchmarks [34] for respondents categorized as having a moderate PSC risk. Next, we found statistically significant differences not only across risk levels in the expected direction but also at a size that can be considered important according to the criterion of minimum 5–10 point for COPSOQ scales used for interpretation of workplace survey results [41]. As expected, we found good reliability of PSC group means when aggregated to the workplace level [46]. Around 15 percentage of the variance was attributed to the workplace, which is a little lower than Dollard and Bakker found in their study [9]. However, 15 percentage can be considered a strong effect [45], and while Dollard and Bakker investigated a more homogenous sample of schools [9], the sample in the current study consisted of a wide variety of workplaces.

We have followed the Australian tradition of using aggregated mean scores for workplaces and applied the same cutoff values for the group level and individual level. However, according to Dollard and Bailey, it is important not only to focus on the average score for the group but also to consider that there might be individuals who are at risk and need attention [12]. An alternative could be to define workplace risk levels based on the proportion of employees being at risk rather than using the aggregated mean score. However, we find it somewhat problematic from a practical as well as an ethical perspective. Usually, workplace risk assessment surveys report mean values for the entire organization and for subgroups of employees (e.g., the way COPSOQ results are reported for the organization, departments, and occupational groups [34]). We believe it is easier to calculate and implement PSC by following this tradition. Besides, reporting the proportion of employees at risk at smaller workplaces may jeopardize the principle of anonymity, which is considered essential when managing psychosocial risks [49]. Previously, a vision that all individuals in an organization should have an individual PSC score indicating low risk has been suggested [31]. If this vision was followed, almost all workplaces would be considered being at risk in the present study. Worth noticing, only a small proportion of employees reporting a low PSC score (indicating a personal high risk) were found at workplaces classified as low risk based on the mean score for all employees.

### 4.4. Strengths and Limitations

Our study has several major strengths. First, it is an advantage that the benchmarks are based on a random sample of employees and therefore representative for employees working in Sweden. Next, the cross-validation of concurrent validity at the workplace level including a test of appropriateness of aggregation of PSC scores was corroborated. This is especially important for practical use of risk assessment at workplaces and for inclusion in multilevel research. Finally, the study builds on a thorough adaptation process using cognitive interviews and cross-cultural validation conducted prior to the present study [21]. 

However, the study also has some limitations which need to be taken into consideration. The cross-sectional study design does not allow causal claims or testing of predictive validity. There is an increased risk of inflated results due to common method bias as the study is based on questionnaire data only. The response rate of the national study was somewhat low, and despite the use of weighting procedures, this might have induced bias. Finally, the workplace sample is not representative of the whole workforce, which limits the possibilities to generalize results regarding the workplace level.

Using central aspects of the Swedish legislative framework for the criteria-based approach while developing the benchmarks for risk levels is an asset. Research from numerous studies have pointed out that PSC is a precursor for several factors linked to both the health deteriorating and the motivating paths of the Job Demands-Resources Model, not merely mental health. Further, it adds to the literature, placing PSC as a true upstream factor since managerial and organizational priority for stress prevention is closely linked to enacting the legislative framework Still, additional validation of prospective validity of PSC in its Swedish version is recommended, for example, how PSC risk levels predict register-based measures such as mental health, sickness absence, or staff turnover.

### 4.5. Implications for Practice and Research

There is evidence that three out of four patients diagnosed with a stress-related exhaustion disorder have decreased stress tolerance and other symptoms 7 years after seeking care [50]. This makes it crucial to find ways of efficiently preventing work-related stress diseases. However, a large-scale regional public sector project reveals that the vast majority of work environment problems are identified to have their roots at the organizational level [51]. Such findings underline the importance of being able to screen workplaces to identify those in most need for organizational level interventions. As such, PSC-4 can be used as a practical tool for monitoring and identifying risk levels of work conditions that can affect employees’ health and productivity. Thus, the short version of PSC could be included in existing projects, e.g., at The Swedish Association of Local Authorities and Regions, or for use as a screening tool for workplace inspections conducted by The Swedish Work Environment Authority. Further, PSC can be used as an evaluation tool for organization-based psychosocial risk prevention and intervention strategies.

Even though research on PSC is increasingly conducted in different countries, there is a need for further studies applying PSC across different cultures and contexts [19,21]. In 2015, Bailey, Dollard, and Richards pointed to a need for future research to ascertain the levels of PSC required in other countries to set standards for worker health [31]. While the principles behind PSC are found to be cross-culturally transferable [21], our finding that the average level of PSC was remarkably lower in Sweden compared to Australia supports the need for more international studies to better understand cultural differences of potential importance determining relevant benchmarks for the local context. For the Swedish context, we recommend further validation of the PSC, the benchmarks, and the suggested use of the instrument as a screening tool. 

## 5. Conclusions

The current study supports the reliability and construct validity of the Swedish version of PSC-4 and establishes benchmarks for PSC risk levels for use in the systematic occupational safety and health management at workplaces. Indications of cultural differences in PSC levels suggest a need for country-specific benchmarks.

## Figures and Tables

**Table 1:** Description of respondents based on a random sample of inhabitants in Sweden aged 25–65 years, gainfully employed (*n* = 2847); Psychosocial Safety Climate (PSC)-4 mean and Standard Deviation (SD); and *p*-values for the difference in PSC-4 mean scores between subgroups based on demographic and work-related characteristics.

Dimension	Group	% Of Total Sample	PSC-4 Mean	PSC-4 SD	*p*-Value
Total sample	*n* = 2847 (weighted data to match target population)	100.0	11.5	4.1	
Gender	Women	56.1	11.4	4.0	0.005
	Men	43.9	11.8	4.0	
Age distribution	25–34 years	15.4	11.3	4.1	0.263
	35–44 years	21.3	11.4	4.2	
	45–54 year	31.3	11.6	4.0	
	55–65 years	32.0	11.7	3.7	
Sector	Private	47.1	11.7	4.1	0.023
	Public	44.7	11.3	3.8	
Weekly work hours	<31 h per week	9.3	11.4	4.1	0.648
	31–40 h per week	55.1	11.5	4.0	
	>40 h per week	33.5	11.6	4.0	
Relational work	Yes	81.1	11.5	4.0	0.369
	No	18.0	11.7	4.0	
Position	Non-managerial position	66.8	11.2	4.0	<0.001
	Managerial position without staff responsibility	16.6	11.7	4.1	
	Managerial position with staff responsibility	16.1	12.8	3.9	
Employment	Fixed position	91.8	11.5	4.0	0.641
	Temporary employment	3.1	11.9	4.0	
	Hourly paid	2.4	11.5	4.1	
Normal work time	Day hours between 6–18 o’clock	78.5	11.7	4.0	<0.001
	Other hours/shiftwork etc.	19.3	10.8	4.2	
Size of local workplace (span of nearest leader)	Up to 10 people	36.7	12.1	4.1	<0.001
	11–20 people	25.1	11.5	3.9	
	21 people or more	34.6	11.0	4.0	

**Table 2:** Multiple regression analysis with PSC-4 (range 1–5) as the dependent variable; separate analyses for men and women, based on the random sample of the Swedish working population (*n* = 2847).

	Men		Women	
	B	*p*	B	*p*
Sector (Public)	0.16	0.018	−0.11	0.078
Position (Managerial)	0.26	0.000	0.17	0.005
Span of nearest leader (<11, 11–20, >21)	−0.12	0.001	−0.11	0.001
Adj r-square	0.03		0.02	
Model *p*	<0.001		<0.001	

**Table 3:** PSC-4 benchmark standards and recommendations.

PSC (4–20)	PSC Standards	Recommendation
>12	Green—Low risk	Continued attention to risk management and further improvement of the organizational and social work environment is recommended.
>8–12	Yellow—Moderate risk	Risk management of the organizational and social work environment needs more attention.
≤8	Red—High risk	Urgent actions are needed for improved management of risks related to the organizational and social work environment.

**Table 4:** For non-managerial employees from the random sample of the Swedish working population (*n* = 1882): mean values and the 95% confidence interval for the scales quantitative demands, quality of leadership, job satisfaction, commitment to the workplace, stress, and burnout (range 0–100) depending on PSC risk level.

Scale	PSC Risk Level	Mean	95% Confidence Interval for Mean	
			Lower Bound	Upper Bound
Quantitative demands ^1^ (national benchmark 40.9)	Low	35.2	33.6	36.7
	Moderate	40.7	39.2	42.3
	High	46.7	44.7	48.8
Quality of Leadership ^1^ (national benchmark 54.1)	Low	68.7	67.2	70.2
	Moderate	53.0	51.6	54.5
	High	34.2	32.2	36.2
Job satisfaction ^1^ (national benchmark 64.4)	Low	72.4	71.2	73.6
	Moderate	64.1	62.9	65.2
	High	48.4	46.6	50.2
Commitment to the Workplace ^1^ (national benchmark 64.7)	Low	78.3	76.9	79.7
	Moderate	64.5	63.0	65.9
	High	42.9	40.9	44.9
Work Engagement ^1^ (national benchmark 69.4)	Low	75.6	74.4	76.8
	Moderate	67.8	66.6	69.1
	High	59.5	57.7	61.4
Stress ^1^ (national benchmark 36.0)	Low	26.1	24.4	27.8
	Moderate	34.4	32.8	36.0
	High	47.2	44.9	49.4
Burnout ^1^ (national benchmark 36.2)	Low	26.0	24.3	27.7
	Moderate	35.7	34.1	37.4
	High	49.1	46.8	51.3

^1^*p*-values for differences in mean score depending on the three PSC risk levels <0.001.

**Table 5:** Intraclass correlation coefficients (ICC(1) and ICC(2)) for aggregation to workplace level (94 workplaces); aggregated mean and standard deviation for PSC-4 (range 1–5); and quantitative demands, quality of leadership, job satisfaction, commitment to the workplace, stress, and burnout (range 0–100) depending on PSC risk level.

	ICC(1) ^2^	ICC(2) ^3^	PSC Risk Level					
			Low		Moderate		High	
			Mean	SD	Mean	SD	Mean	SD
PSC-4 ^1^	0.15	0.86	13.3	1.1	10.5	0.9	7.6	0.2
Quantitative Demands ^1^	0.19	0.88	39.1	10.2	41.9	10.1	58.8	3.3
Quality of Leadership ^1^	0.15	0.85	64.6	10.5	55.7	10.6	38.3	5.3
Commitment to the Workplace ^1^	0.12	0.82	70.8	9.9	59.4	8.8	43.5	10.2
Job Satisfaction ^1^	0.07	0.72	69.6	5.7	63.6	5.6	52.1	1.6
Work Engagement	0.05	0.62	72.6	5.5	69.3	7.2	72.7	4.4
Stress ^1^	0.05	0.65	27.7	6.8	33.5	7.4	46.1	6.0
Burnout ^1^	0.06	0.68	29.2	6.9	35.9	6.5	48.8	0.2

^1^*p*-values for differences in mean score depending on the three PSC risk levels <0.001. ^2^ ICC(1) represents the amount of variance in the employees’ responses that can be explained by the membership of their workplace ^3^ ICC(2) is an estimate of reliability of the aggregated group means.

## Appendix A

Overview of items covering OSH practices and PSC-4, item intercorrelations, and factor analysis.

**Table A1:** Item formulations in English and Swedish for Occupational Safety and Health (OSH) practices and PSC items.

	Item in English	Item in Swedish	Response Option	Origin of Item
OSH1	Is it clear who is responsible for work environment issues in your workplace?	Är det klart och tydligt vem som har ansvar för arbetsmiljöarbetet på din arbetsplats?	*	1
OSH2	Does your employer regularly examine working conditions and assess the risks of illness or accidents at work?	Undersöker och bedömer din arbetsgivare regelbundet riskerna för ohälsa och olycksfall i arbetet?	*	1
OSH3	Does your employer immediately or as soon as possible implement the measures needed to prevent illness and accidents?	Genomför din arbetsgivare så snart det är praktiskt möjligt de åtgärder som behövs för att förebygga ohälsa och olycksfall?	*	1
OSH4	Are there procedures how to handle victimization in your organization?	Finns rutiner för hantering av kränkande särbehandling inom din organisation?	*	2
OSH5	Is your employer fully committed to creating healthy working conditions?	Arbetar din arbetsgivare målmedvetet för att skapa hälsosamma arbetsvillkor?	*	2
PSC3	Senior management shows support for stress prevention through involvement and commitment.	Högsta ledningen stödjer stressförebyggande arbete i organisationen	**	3
PSC6	Senior management considers employee psychological health to be as important as productivity.	Högsta ledningen anser att medarbetarnas psykiska hälsa är lika viktigt som organisationens prestationsmål	**	3
PSC7	There is good communication here about psychological safety issues which affect me.	Det finns en bra kommunikation om psykosociala säkerhetsfrågor bland medarbetarna i vår organisation	**	3
PSC12	In my organization, the prevention of stress involves all levels of the organization.	Stressförebyggande arbete involverar samtliga nivåer i vår organisation	**	3

* Yes; no; do not know/ja; nej; vet inte. ** Strongly disagree; disagree; neither agree or disagree; agree; strongly agree/instämmer inte alls; instämmer i låg grad; varken instämmer eller är emot; instämmer i hög grad; instämmer helt 1. Based on the Swedish provisions for organizational and social work environment (AFS 2015:4Eng) and formulations inspired by questions included in The Swedish Work Environment Authority’s biannual survey [13,38]. 2. Proprietary item based on the Swedish provisions for organizational and social work environment (AFS 2015:4Eng). 3. [14,21,36,37] (copyright is at maureen.dollard@unisa.edu.au).

**Table A2:** Bivariate Spearman’s Rho correlations between the items covering OSH practices and PSC.

Items	OSH1	OSH2	OSH3	OSH4	OSH5	PSC3	PSC6	PSC7	PSC12
OSH1	1								
OSH2	0.42 **	1							
OSH3	0.41 **	0.54 **	1						
OSH4	0.30 **	0.33 **	0.30 **	1					
OSH5	0.38 **	0.43 **	0.51 **	0.35 **	1				
PSC3	−0.25 **	−0.24 **	−0.31 **	−0.23 **	−0.42 **	1			
PSC6	−0.28 **	−0.22 **	−0.31 **	−0.21 **	−0.41 **	0.79 **	1		
PSC7	−0.31 **	−0.27 **	−0.34 **	−0.25 **	−0.42 **	0.69 **	0.73 **	1	
PSC12	−0.26 **	−0.25 **	−0.30 **	−0.23 **	−0.43 **	0.76 **	0.76 **	0.78 **	1

** Correlation is significant at the 0.01 level (2-tailed).

**Table A3:** Rotated pattern matrix factor loadings for factor analysis with direct oblimin rotation for two components.

Items	Rotated Component Coefficients	
	Component 1	Component 2
OSH1	0.02	**0.68**
OSH2	0.10	**0.82**
OSH3	−0.01	**0.78**
OSH4	−0.01	**0.59**
OSH5	−0.22	**0.63**
PSC3	**0.91**	0.02
PSC6	**0.92**	0.03
PSC7	**0.87**	−0.06
PSC12	**0.92**	0.00

Note. Major loadings for each item are bolded.

## Appendix B

Overview of response profiles of OSH practices based on non-managerial employees from the random sample of the Swedish working population.

**Table A4:** Classification of OSH practices according to the criterion based approach (CBA) into good practices, fair practices, and poor practices based on the number of responses found in the response categories yes, don’t know, and no out of 5 OSH items linked to the Swedish regulations: for each response combination, the PSC mean and standard deviation as well as number of respondents is presented, based on a random sample of non-managerial employees Swedish working population (*n* = 1862).

Response Combination	Yes	Don’t Know	No	CBA OSH- Practices	PSC Mean	PSC SD	*n*
(1)	5	0	0	Good	14.1	3.2	490
(2)	4	1	0	Good	12.6	3.0	183
(3)	4	0	1	Good	11.4	4.0	50
(4)	3	2	0	Good	12.2	3.1	150
(5)	3	1	1	Fair	11.0	3.3	73
(6)	3	0	2	Fair	10.6	3.4	50
(7)	2	3	0	Fair	11.0	3.4	106
(8)	2	2	1	Fair	10.0	2.7	67
(9)	2	1	2	Fair	9.9	3.0	39
(10)	1	4	0	Fair	11.0	3.0	81
(11)	1	3	1	Fair	9.6	3.3	63
(12)	0	5	0	Fair	10.2	3.2	80
(13)	0	4	1	Fair	9.9	3.1	45
(14)	1	2	2	Fair	8.2	3.1	45
(15)	0	3	2	Poor	9.2	2.9	43
(16)	2	0	3	Poor	8.3	3.5	47
(17)	1	1	3	Poor	8.1	3.8	39
(18)	0	2	3	Poor	8.0	2.9	48
(19)	1	0	4	Poor	7.8	3.2	43
(20)	0	1	4	Poor	6.7	3.0	37
(21)	0	0	5	Poor	6.6	3.0	83

**Table A5:** Distribution of responses, mean, standard deviation (SD), and 95% confidence interval (CI) for mean of PSC-4 (range 4–20) depending on OSH practices, based on a random sample of non-managerial employees from the Swedish working population (*n* = 1882).

OSH-Practices	Response Distribution	Mean	SD	95% CI for Mean	
Good	47%	13.3	3.4	13.1	13.5
Fair	35%	10.2	3.2	10.0	10.5
Poor	18%	7.6	3.3	7.3	8.0

## Appendix C

PSC item response distribution for the random sample of the Swedish working population.

**Table A6:** Distribution of item responses in percentages and Cronbach’s alpha if the item was deleted from the scale (managerial and non-managerial employees).

Item	Strongly Disagree	Disagree	Neither Agree or Disagree	Agree	Strongly Agree	Missing	Cronbach’s Alpha if Item Deleted
PSC3	12.2	18.5	39.1	20.9	8.2	1.2	0.91
PSC6	13.3	18.2	34.8	22.0	10.3	1.5	0.91
PSC7	13.8	19.4	38.2	21.0	6.0	1.6	0.91
PSC12	17.5	20.7	38.6	15.0	6.3	1.9	0.90
